# GeneXpert MTB/RIF Ultra in Pediatric Tuberculosis: How Disease Characteristics Modify Test Performances

**DOI:** 10.2174/0115733963324694241008081106

**Published:** 2025-01-06

**Authors:** Domenico Pace, Roberta Pellegrino, Irene Dalpiaz, Marco Renni, Luisa Galli, Elena Chiappini

**Affiliations:** 1 Department of Health Sciences, University of Florence, Florence, Italy;; 2 Infectious Disease Unit, Meyer Children’s Hospital IRCCS, Florence, Italy

**Keywords:** Children, GeneXpert Ultra, sensitivity, specimen, tuberculosis, diagnosis

## Abstract

**Introduction:**

The diagnosis of pediatric tuberculosis (TB) is challenging, due to the lower sensitivity of microbiological tests, such as culture and microscopy, compared to their performance in adult cases. Guidelines have introduced molecular tests, including GeneXpert MTB/RIF and GeneXpert MTB/RIF Ultra. These tests use a real-time polymerase chain reaction method and provide information on *M. tuberculosis* detection and drug-resistance-associated mutations in less than 2 hours. This retrospective single-center study aimed to evaluate the accuracy of GeneXpert and GeneXpert Ultra for the diagnosis of pediatric TB.

**Methods:**

This retrospective study was conducted on a total of 95 children diagnosed with probable or confirmed TB disease (74 diagnosed with pulmonary TB, 21 with extrapulmonary TB), who referred to the infectious disease unit at Meyer Children’s Hospital in Florence, Italy, and tested with GeneXpert MTB/RIF or GeneXpert Ultra, from 2013 to 2023.

**Results:**

GeneXpert and GeneXpert Ultra demonstrated a detection rate of 0.357 (95% CI 0.180 to 0.535) and 0.537 (95% CI 0.417-0.657), respectively. No child was tested with both tests. Patients’ characteristics, including age and sex, did not significantly influence the test's performances. Notably, GeneXpert Ultra had a significantly higher detection rate in children with extrapulmonary TB (0.813, 95% CI 0.621 to 1.004) compared to that in children with pulmonary TB (*p* = 0.020). Gastric aspirate was the most tested specimen. Specimens that did not require invasive procedures for collection (including stool) yielded poor results. GeneXpert and GeneXpert Ultra permitted rapid evaluation of genotypic drug-sensitivity testing (DST), even though limited to rifampicin resistance, making necessary confirmation through phenotypic DST (performed on culture).

**Conclusion:**

The introduction of GeneXpert and GeneXpert Ultra improved TB diagnosis in children, by providing microbiological information in a short time, complementing results from culture, which remains the reference test in pediatric TB diagnosis.

## 1 INTRODUCTION

Pediatric tuberculosis (TB) had an incidence rate of 1.3 million cases and a mortality rate of 214,000 children in 2022 worldwide [1], even though it is a disease often neglected [2, 3]. It requires different management since the presentation and characteristics of TB in children differ slightly from those in adults [4-6]. Peculiarities of pediatric TB include the higher risk of extrapulmonary and disseminated disease [7, 8] and the challenge in microbiological diagnosis [9-12]: children often present with a paucibacillary form of the disease, characterized by a lower number of bacilli in specimens [4, 5, 13]. Additionally, collecting specimens can be difficult in pediatric patients, particularly in younger children [14, 15], who may not be able to expectorate [16, 17], who may not tolerate collection methods [18], and whose caregivers may not accept invasive collection techniques [19]. Studies and guidelines suggest the collection of specimens such as stool and urine, that do not require invasive collection and seem to be associated with encouraging results [20-25]. For these reasons, microbiological tests that consent to the definite diagnosis of TB are associated with lower sensitivities in children: a 2016 review reported a culture sensitivity of 0.244 (IQR 0.156-0.424) [26]. A 2016 review on microscopy highlighted the difference between the sensitivity in children and adults: 6.8% (95% CI 2.2% to 12.2%) and 52.0% (95% CI 40.0% to 64.0%), respectively [27].

When microbiological tests yield negative results in children with a high probability of TB, diagnosis relies solely on clinical and radiological characteristics: these diagnoses are referred to as unconfirmed/probable TB [28].

The lower sensitivity of microbiological tests [29] also limits the evaluation of drug-susceptibility testing (DST), which is considered fundamental to setting up appropriate antitubercular treatment [19]. When DST results are not available, children are administered empirical treatment, and the choice of drugs is based on guidelines, drug-sensitivity pattern of the contact case (when identified), drug-resistance incidence in the country of origin, and response to treatment [19].

Since 2013, WHO guidelines have recommended GeneXpert MTB/RIF and GeneXpert Ultra as initial diagnostic assays [1]. GeneXpert MTB/RIF is a cartridge-based automated test that uses real-time polymerase chain reaction on the GeneXpert platform to identify *M. tuberculosis* complex and mutations associated with rifampicin resistance [30-33]. GeneXpert Ultra, conducted with a new cartridge on the same platform, enhances sensitivity and reliability [30, 34, 35]. Data suggest that both GeneXpert Ultra [36] and GeneXpert [37] demonstrated good sensitivity in children. A 2022 Cochrane review reported a sensitivity of 70.4% (95% CI 53.9 to 82.9) for GeneXpert Ultra on gastric aspirate in culture-confirmed cases [38], while a 2020 Cochrane review reported a sensitivity of 73.0% (95% CI 52.9% to 86.7%) for GeneXpert [39]. GeneXpert Ultra can also give trace results, common in pediatric specimens, of the paucibacillary nature of TB disease [40]. For children and people living with HIV being evaluated for pulmonary TB (PTB), and for people being evaluated for Extrapulmonary TB (EPTB), the “*M. tuberculosis* complex detected trace” Ultra result is considered as bacteriological confirmation of TB [30]. These tests give a response in less than 2 hours [30], a significant improvement over the 8 weeks required for culture to yield negative results [4]. GeneXpert MTB/RIF and GeneXpert Ultra also permit rapid genotypic DST, evaluating drug-resistance-associated mutations [41-44].

WHO recommends using sputum, gastric aspirate, nasopharyngeal aspirate, and stool as specimens for PTB diagnosis in children when employing GeneXpert or GeneXpert Ultra [19]. The collection and testing of different specimens improves test performances [45]. Stool recently considered for PTB diagnosis, is an accessible specimen, in particular in younger children [15, 30, 46-48].

This retrospective single-center study aimed to evaluate the accuracy of GeneXpert and GeneXpert Ultra for the diagnosis of pediatric TB and for the evaluation of genotypic DST in a low-burden area, studying how children's characteristics, disease characteristics and specimens influence the performance of these tests.

## 2 MATERIALS AND METHODS

### 2.1 Selection Criteria

We conducted a retrospective observational study including all children, aged <18 years, consecutively referred for suspected TB to the Pediatric Infectious Disease Unit (IDU) of Meyer Children’s University Hospital, Florence, Italy, from 2013 to 2023, and that were tested with GeneXpert or GeneXpert Ultra. No child was tested with both tests: from January 2013 to April 2017, children were tested with GeneXpert, while starting from May 2017, GeneXpert Ultra was adopted. Children included in the study were part of a larger group referred to the IDU due to clinical suspicion of TB disease and/or close contact with recently diagnosed cases of contagious TB disease and/or being internationally adopted or recently immigrated children coming from countries with high prevalence of TB.

The clinical suspicion of TB was based on the presence of signs and symptoms suggestive of TB (fever, weight loss or poor weight gain, cough, night sweats, hemoptysis), chest imaging findings (lymphadenopathy of the hilar, subcarinal, paratracheal, or mediastinal nodes; atelectasis or infiltrate of a segment or lobe; pleural effusion; cavitary lesions), positive tuberculin skin test (TST) or interferon-gamma release assays (IGRAs), close contact with an infectious TB source case within the preceding three months, or history of international adoption or recent immigration from countries with a high prevalence of TB [23, 30, 39].

### 2.2 Data Collection

For each child included in the study, the following information was collected and entered into an electronic database: demographic characteristics (sex, age at first admission, place of birth, nationality); the reason for TB investigation and personal history of previous TB contact; TST and IGRAs results; microbiological investigations results (culture, microscopy, GeneXpert or GeneXpert Ultra) and TB drug sensitivity profiles.

The study has been conducted and reported according to STROBE guidelines for observational studies [49].

### 2.3 Diagnosis and Laboratory Tests

At first examination, all children underwent clinical evaluation, TST and venipuncture for the IGRA assay QuantiFERON-Gold In-Tube (QFT-IT) [19, 50-52]. Chest radiography was performed in all symptomatic children [53], those with a positive TST/QTF-IT and in all contacts aged less than 5 years. Chest computer tomography (CT) scans were performed in selected cases at the discretion of the pediatrician [22, 54-56]. This screening allowed an initial categorization of children into three groups: 1) not-infected children (including those with negative TST/QTF-IT test and negative chest X-ray/CT scan); 2) tuberculosis infection (TBI) (including children with positive TST/QTF-IT and negative chest X-ray/CT scan); 3) children with suspected pulmonary and/or extra-pulmonary TB.

Children with suspected PTB or EPTB and tested with GeneXpert or GeneXpert Ultra were eligible for the study. They were also tested with microscopy and culture according to National and International Guidelines recommendations [30, 39].

Children were classified as having active TB, TBI, and not infected, according to the American Academy of Pediatrics (AAP) guidelines [57]. Cases of active TB were defined in two categories:

• Confirmed TB: children with confirmation of *M. tuberculosis* obtained from culture, or GeneXpert/GeneXpert Ultra.

• Probable TB: children without microbiological confirmation of TB but presenting with at least two of the following criteria: signs and symptoms suggestive of TB; radiological findings consistent with TB; positive response to TB treatment; immunological evidence of *M. tuberculosis* infection (TST and/or QTF-IT positive); either a history of TB contact or travel to a TB-endemic country within the last 24 months [28].

According to AAP and national guidelines, TBI diagnosis was assigned to any child with a positive TST or QFT-IT and no clinical, bacteriological, or radiographic evidence of active TB [57]. Children without bacteriological confirmation and without criteria for probable TB or TBI were considered not infected [57]. The appropriate size of induration indicating a positive TST result was established according to the national and international guidelines:

• 15 mm or greater in children without any risk factor.

• 10 mm or greater in children at increased risk of disseminated TB disease.

• 5 mm or greater in children in close contact with contagious people with TB, in children suspected to have TB disease (due to radiograph and clinical evidence), in children receiving immunosuppressive therapy or with immunosuppressive conditions [57].

Gastric lavage aspirate (GLA) samples were collected in the morning after overnight fasting. Through a nasogastric tube, 10 mL of saline solution, and then a minimum of 5 mL of aspirate was obtained [58]. Tests were conducted on 3 gastric samples [59, 60]. GLA samples were processed as described previously [58].

On each sample, smear microscopy, culture and GeneXpert/GeneXpert Ultra were performed. Smear microscopy was performed through fluorescence microscopy on fluidized, decontaminated, concentrated, fixed and auramine-rhodamine colored samples. Samples were inoculated on solid *Lowenstein-*Jensen (LJ) media for up to 60 days and on liquid BACTEC Mycobacterium Growth Indicator Tube 960 (MGIT) liquid culture for up to 42 days. On positive cultures, Line Probe Assay (LPA) and phenotypic drug susceptibility testing (DST) were performed with the BD MGIT™ TBC Identification Test.

Specimens different from GLA were tested, including sputum, broncho-alveolar lavage, biopsy, stool, urine, ascites, pleural fluid, cerebrospinal fluid, synovial fluid, and purulent material [38]. The collection of determined specimens was led by clinical presentation, risk of extrapulmonary/diffuse TB and clinicians’ choices. Non-respiratory specimens were mostly collected in cases with suspicion of extrapulmonary disease and in cases at high risk of disseminated disease.

GeneXpert and GeneXpert Ultra were performed according to the manufacturer’s instructions [58]. Results were generated in 2 hours and reported as MTB-negative or MTB- positive with a semi-quantified bacillary load as high, medium, intermediate, low, or trace. The assay tested, at the same time, rifampicin sensitivity.

### 2.4 Statistical Methods

Children were categorized for age (≤5 years old, >5 years old), origins (Italian origins, foreign origins), reasons for investigation (children who had contact with a case, children with symptoms), BCG vaccination (vaccinated, not vaccinated), and disease location (PTB, EPTB).

Data were reported as median and interquartile range (IQR) or absolute numbers and percentages. Fisher’s exact test or Chi-Square test with Yates correction was used to compare categorical variables when appropriate.

The detection rate of GeneXpert MTB/RIF, GeneXpert Ultra, culture and microscopy were established with 95% confidence intervals (CIs). The results of culture, microscopy, GeneXpert MTB/RIF and GeneXpert Ultra were compared with clinical diagnosis of TB as the reference standard.

*P* <0.05 was considered statistically significant. Statistical analysis was performed using the Stata Software, version 14.0 (StataLLC, College Station, TX) for Windows.

## 3 RESULTS

The study included 95 children (median age 6 years, IQR 2-13) diagnosed with confirmed or probable TB and tested with either GeneXpert or GeneXpert Ultra: 28 children (29.47%) were tested with GeneXpert and 67 (70.53%) children were tested with GeneXpert Ultra. Their characteristics are summarized in Tables **1** and **2**.

GeneXpert was positive in 10/28 children, resulting in a detection rate of 0.357 (95% CI 0.180 to 0.535). GeneXpert Ultra was positive in 36/67 children, resulting in a detection rate of 0.537 (95% CI 0.417-0.657). The results of GeneXpert and GeneXpert Ultra did not show a significant difference (*p* = 0.168).

When considering only culture-confirmed diagnosis, GeneXpert had a detection rate of 0.389 (95% CI 0.164 to 0.614), and GeneXpert Ultra of 0.757 (95% CI 0.618 to 0.895), resulting in significantly different (*p* = 0.018).

The performances of tests were also evaluated considering case characteristics.

### 3.1 Age and Origins

In children aged ≤5 years old, GeneXpert had a detection rate of 0.308 and GeneXpert Ultra of 0.500. In children aged >5 years old, GeneXpert had a detection rate of 0.400 and GeneXpert Ultra of 0.576. No significant difference was found between the results of children aged ≤5 years old and >5 years old in both tests (Tables **1** and **2**).

There were 24 (25.26%) children born in Italy to Italian parents and 71 (74.74%) of foreign origins (14 from Eastern Europe, 29 from Africa, 18 from Asia, and 10 from Central- South America). GeneXpert yielded positive results in 2/5 (0.400) Italian children and 8/23 (0.348) foreign children (not significantly different detection rate, *p* = 1.000). GeneXpert Ultra sensitivity was 0.316 in Italian children and 0.625 in foreign children, resulting in significantly higher *p* = 0.044.

The comparisons of GeneXpert and GeneXpert Ultra results in these classes of patients and their CIs are detailed in Table **3**. The performances of the two tests did not result significantly different in any of the studied characteristics.

### 3.2 Reasons for Investigation, TST/IGRAs Positivity and Disease Location

The children were diagnosed with TB after contact with a case in 47 cases (49.47%), after presenting symptoms in 43 cases (45.26%), and as part of the screening for immigrated/adopted children of the hospital in 5 cases (5.26%). GeneXpert was associated with a detection rate of 0.462 in children diagnosed after a contact, of 0.333 in children with symptoms, while 0/3 children were positive in screening. GeneXpert detection rates were not significantly different for any of these classes of patients (Table **1**). GeneXpert Ultra was associated with a detection rate of 0.412 in children diagnosed after a contact, 0.645 in children with symptoms, and 2/2 children were positive in screening. GeneXpert Ultra results were not significantly different for any of these classes of patients (Table **2**).

The diagnosis of infection was achieved through TST and/or IGRAs. GeneXpert had a detection rate of 0.320 in children positive for TST/IGRAs and 0.667 in children negative for TST/IGRAs. These results were not significantly different (Table **1**). GeneXpert Ultra had a detection rate of 0.556 in children positive for TST/IGRAs and 0.250 in children negative for TST/IGRAs. These results were not significantly different (Table **2**).

In children with a diagnosis of PTB, GeneXpert had a detection rate of 0.348, and in children with EPTB of 0.400. GeneXpert detection rates were not significantly different (Table **1**). In children with a diagnosis of PTB, GeneXpert Ultra had a detection rate of 0.451, and in children with EPTB of 0.813. The results of GeneXpert Ultra in PTB and EPTB were significantly different (*p* = 0.020, Table **2**).

The comparisons of GeneXpert and GeneXpert Ultra detection rates in the classes of patients described and their CIs are detailed in Table **4**. The detection rates of the two tests did not result significantly in any of the studied characteristics.

### 3.3 Specimens

**Fig. (1):**
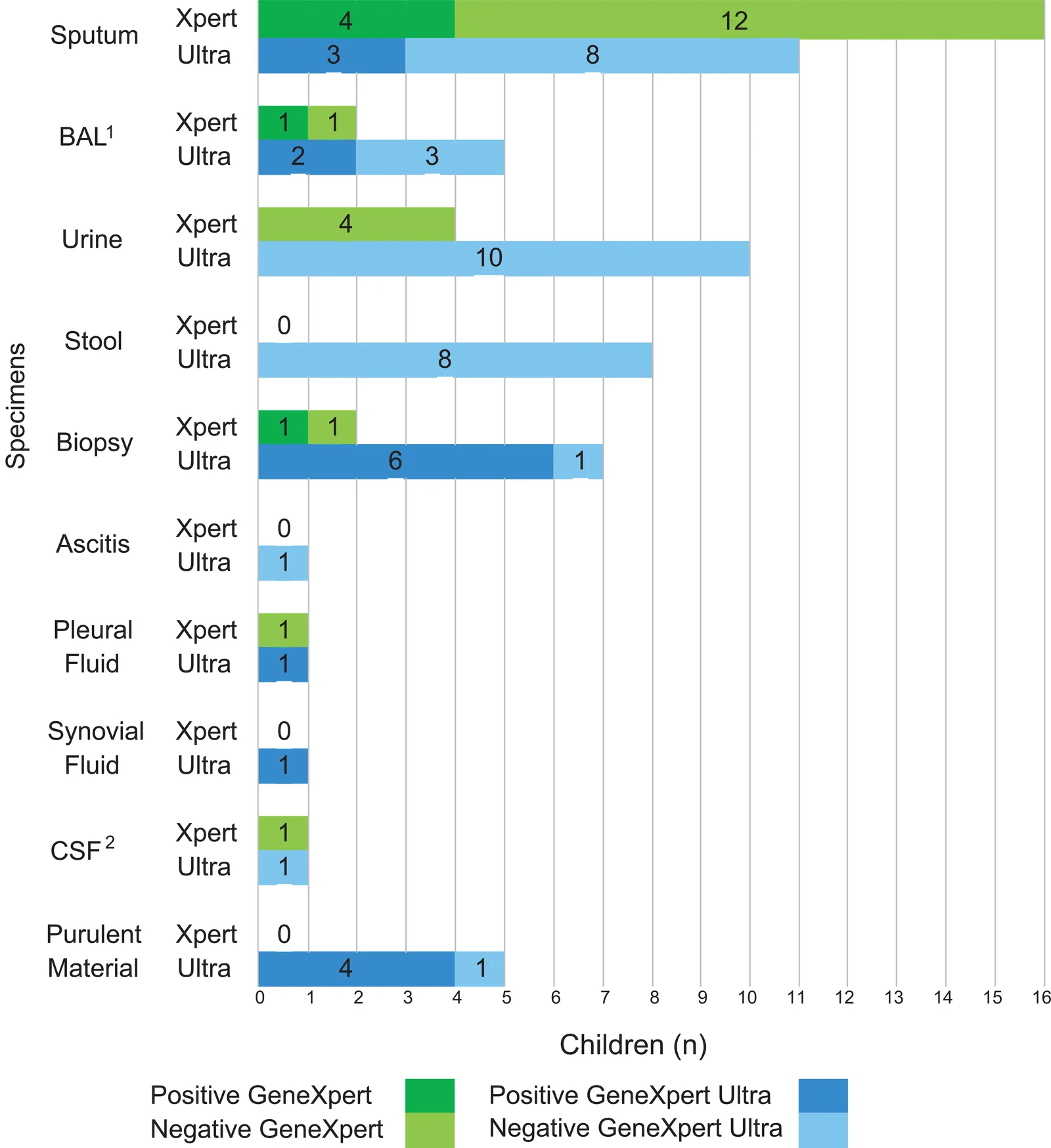
Specimens different from gastric aspirate tested, and the number of tests and positive results associated with each specimen. ^1^ Broncho-Alveolar Lavage ^2^ Cerebrospinal fluid.

GeneXpert and GeneXpert Ultra were conducted on different specimens (Table **5**, Fig. **1**). In GeneXpert, 18/28 (64.29%) children were tested on one specimen, 10/28 (35.71%) on two or more specimens. In GeneXpert Ultra, 37/67 (55.22%) children were tested on one specimen, 30/67 (44.78%) on two or more specimens.

GeneXpert has been conducted on gastric aspirate in 14/28 children (50.00%) and had a detection rate of 0.429 (95% CI 0.169 to 0.688). GeneXpert conducted on specimens different from gastric aspirate had a detection rate of 0.231 (95% CI 0.069 to 0.393). These results were not significantly different (*p* = 0.347).

GeneXpert Ultra has been conducted on gastric aspirate in 64/67 children (95.52%) and had a detection rate of 0.391 (95% CI 0.271 to 0.510). GeneXpert Ultra conducted on specimens different from gastric aspirate had a detection rate of 0.321 (95% CI 0.195 to 0.446). These results were not significantly different (*p* = 0.555).

GeneXpert and GeneXpert Ultra results were not significantly different, both on gastric aspirate (*p* = 0.969) and other specimens (*p* = 0.573).

### 3.4 Drug Sensitivity

Among children who tested positive in GeneXpert, 10/10 (100.00%) exhibited rifampicin sensitivity. None of them had drug-resistant phenotypic DST.

Among children who were positive in GeneXpert Ultra, 30/36 (83.33%) had rifampicin sensitivvity (1/30 had mono-resistant to isoniazid in phenotypic DST), 1/36 (2.78%) was rifampicin-resistant (confirmed in phenotypic DST, which also revealed streptomycin, isoniazid, pyrazinamide and ethambutol resistances), and 5/36 (13.89%) with undetermined mutations (1/5 were isoniazid-resistant in phenotypic DST). Moreover, 2 children negative in GeneXpert Ultra were pyrazinamide resistant in phenotypic DST.

### 3.5 Other Diagnostic Tests Results

Culture and microscopy results are reported in Tables **6** and **7**. The culture was positive in 55/95 children (detection rate 0.579, 95% CI 0.480 to 0.678). The culture detection rate was not significantly different when compared to GeneXpert (0.357, *p* = 0.064) and GeneXpert Ultra (0.537, *p* = 0.715).

Among children positive in GeneXpert, 3 were negative in culture: 2 children were Italian, diagnosed with PTB and were ≤5 years old; 1 child originated from Asia, was diagnosed with EPTB and was >5 years old. They were all diagnosed after contact with a case and had a diagnosis of infection through TST and/or IGRAs. Among children positive in GeneXpert Ultra, 8 resulted negative in culture; their characteristics are summarized in Fig. (**2**). They had a diagnosis of infection through TST and/or IGRAs.

GeneXpert negative results were 18, and GeneXpert Ultra negative results were 31. Negative results were compared with culture: 11 GeneXpert negative cases resulted in culture positive, and 9 GeneXpert Ultra negative cases resulted in culture positive.

Ultimately, 29 children (30.53%) had probable TB diagnosis, being culture, microscopy and GeneXpert (7/28) or GeneXpert Ultra (22/67) negative. In these cases, the diagnosis was not supported by microbiological confirmation.

## 4 DISCUSSION

This retrospective study evaluated the performance of GeneXpert and GeneXpert Ultra in children diagnosed with confirmed TB or probable TB. The tests demonstrated detection rates comparable to those reported in the literature. GeneXpert Ultra showed better performances in children with a diagnosis of EPTB. The gastric aspirate was the most tested specimen. Specimens that do not require invasive procedures to be collected were not associated with satisfying test performances. GeneXpert and GeneXpert Ultra helped in the rapid evaluation of drug-sensitivity, even though they did not identify one MDR-TB case since they evaluated only rifampicin-associated mutations.

Considering only confirmed TB cases, GeneXpert Ultra performances approached values reported in the literature, while GeneXpert had lower results: Kaur *et al.* reported a sensitivity of 64.29% (95% CI 51.93% to 75.93%) for GeneXpert and a sensitivity of 80% (95% CI 68.73% to 88.61%) for GeneXpert Ultra, in a cohort of 130 children younger than 15 years [36]; Chakravorty *et al.* reported 81.0% (95% CI 74.9% to 86.2%) for GeneXpert, and 87.5% (95% CI 82.1% to 91.7%) for GeneXpert Ultra [61]. Various studies reported GeneXpert Ultra sensitivity being higher than GeneXpert sensitivity [34, 39, 62-64]. In our study, the detection rates are significantly different when considering only confirmed TB cases, not when considering also patients with probable TB. When calculated considering all the children included in the study (diagnosed with probable TB or confirmed TB), the detection rate of GeneXpert and GeneXpert Ultra resulted in lower than values reported in the literature. Nevertheless, GeneXpert Ultra exhibited a detection rate higher than 0.500 also in our population of patients.

GeneXpert Ultra performed significantly better in EPTB cases. Aurilio *et al.* reported that GeneXpert Ultra yielded positive results in 77% of children diagnosed with EPTB [65]; moreover, they described GeneXpert Ultra's usefulness in pleural TB diagnosis [65], while in our study, pleural fluid was tested with GeneXpert Ultra in one case only, resulting positive. Pradhan *et al.* highlighted the role of GeneXpert Ultra in tuberculous meningitis diagnosis [66], a result that our study was not able to confirm, considering that CSF was tested with GeneXpert Ultra in only one child. GeneXpert Ultra is capable of yielding “trace” results, when detecting a very low quantity of *M. tuberculosis* DNA [61, 67, 68]. This probably permits GeneXpert Ultra to better diagnose EPTB cases that often present with paucibacillary specimens [69-71]. Given the challenges in TB diagnosis in children [4] and the higher prevalence of EPTB and severe forms in younger children [72], it would be useful to investigate these results in a review, considering that in single-center studies, these cohorts of patients are usually small.

Gastric aspirate was the most tested specimen; its sensitivities in GeneXpert and GeneXpert Ultra were comparable to results reported by Kay *et al.* in their 2020 and 2022 reviews: GeneXpert had a sensitivity on gastric aspirate of 0.317 (95% CI 0.202 to 0.460) and 0.730 (95% CI 0.529 to 0.867) in culture confirmed cases [39]; GeneXpert Ultra had a sensitivity of 0.704 (95% CI 0.539 to 0.829) and 0.465 (95% CI 0.297 to 0.641) in culture confirmed cases [38] Specimens that require invasive procedures to be collected, such as BAL and biopsy, were tested in a small number of children but were associated with a high percentage of positive results. However, stool (suggested in WHO guidelines [19]) and urine specimens easily collected without invasive procedures were associated with no positive results. It must be noted that other studies reported more promising results: GeneXpert Ultra tested on the stool had a sensitivity of 0.561 (95% CI 0.391 to 0.717) in the 2022 Kay **et al.*’s* review [38], was positive in 83.3% of children with PTB (13.4% considering also probable PTB diagnosis) in Kabir **et al.*’s* article [73]. In Sun **et al.*’s* GeneXpert Ultra on stool (60.3%, 85/141) and on GLA (52.5%, 74/141) were associated with similar sensitivities (p = 0.187) [74]. Probably, these specimens were not ideal in a low-burden setting, as ours. Data suggest that testing culture and GeneXpert/GeneXpert Ultra together are beneficial.

Culture identified 20 cases of TB that resulted negative with GeneXpert/GeneXpert Ultra, while GeneXpert/GeneXpert Ultra identified 11 cases that resulted negative in culture. Combining these tests can help to identify cases missed by either method alone, enhancing diagnostic accuracy [60]. Furthermore, genotypic DST was less complete compared with phenotypic DST, even though the development and application of new PCR-based technologies could reduce this gap [75].

Limitations in our study included a not large study population (and subsequently few samples tested), being a single center study held in a low-burden setting, the low number of children with EPTB (a class of patients interesting to analyze, but not as frequent as PTB); the fact that extrapulmonary specimens were tested in a limited number of children; children had different samples tested; finally, having included only cases diagnosed with TB disease, it was not possible to discuss GeneXpert and GeneXpert Ultra false positive results.

## CONCLUSION

GeneXpert and GeneXpert Ultra introduction improved TB diagnosis in children, providing microbiological information in a short time. GeneXpert Ultra was associated with better performances in EPTB cases, confirming its importance in identifying cases that are often difficult to diagnose. In our setting, specimens that do not require invasive procedures to be collected were not associated with encouraging performances, even though the single-center nature of our study invites caution. Despite the longer time required by culture, it remains the reference test, also considering the larger information provided by phenotypic DST compared with genotypic DST. The development of new PCR-based technologies could reduce this gap in the near future.

## Figures and Tables

**Fig. (2):**
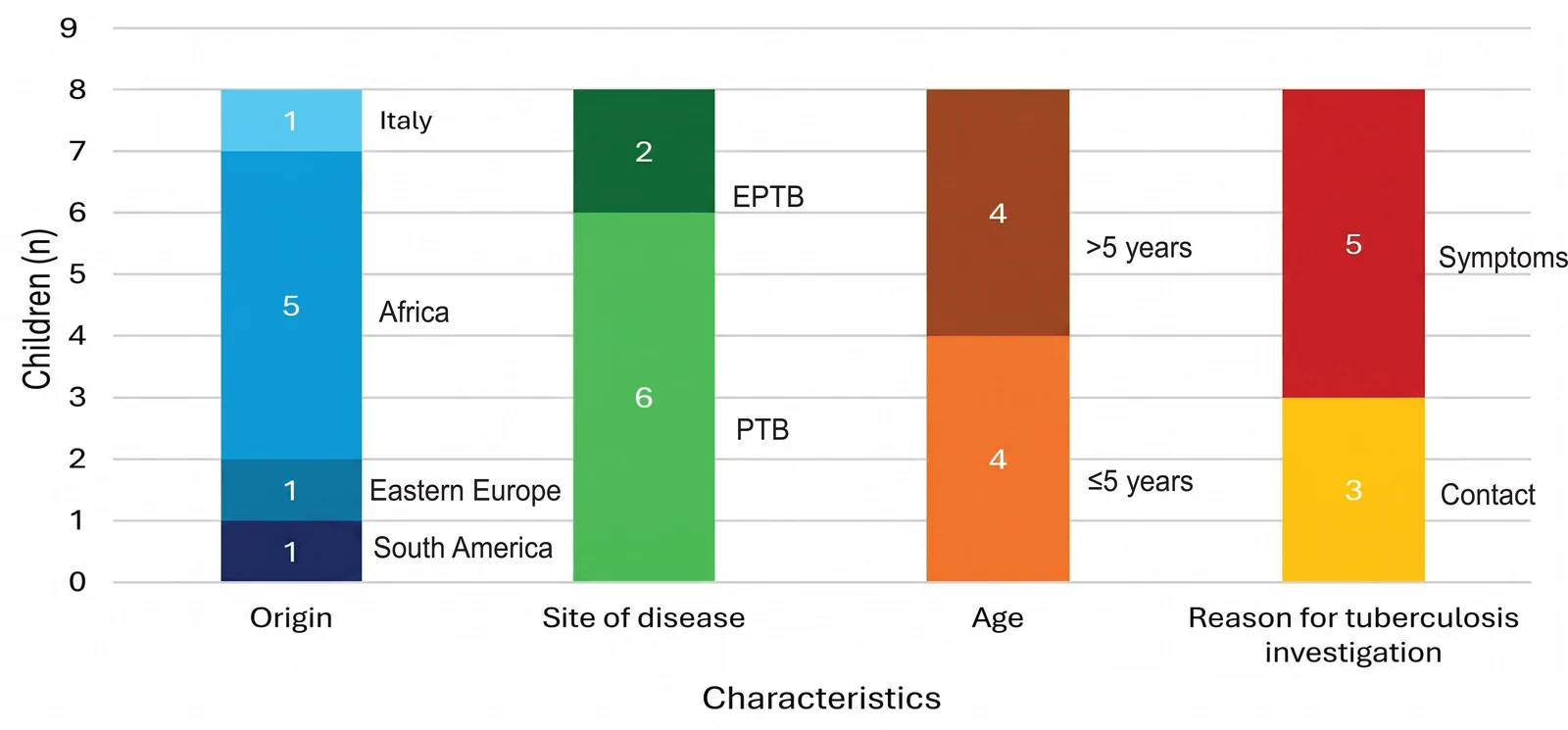
Characteristics of children with negative culture and positive GeneXpert Ultra, considering origin, disease location, age and reason for investigation. ^1^ Extrapulmonary tuberculosis ^2^ Pulmonary tuberculosis.

**Table 1:** Characteristics of cases tested with GeneXpert MTB/RIF and p-value evaluating GeneXpert MTB/RIF performances among different groups of children. ^1^ Children born in Italy or abroad to non-Italian parents. ^2^ Children diagnosed during the screening for immigrated and adopted children held by the IDU.^3^ Bacillus Calmette-Guerin. ^4^ Tuberculin Skin Test.

**Study Children Characteristics**	**Children who Tested Positive in GeneXpert**	**Children who Tested Negative in GeneXpert**	**Total**	***p***
	**n = 10 (35.71%)**	**n = 18 (64.29%)**	**n = 28 (%)**	-
Male	6 (60.00)	10 (55.56)	16 (57.14)	1.000
Female	4 (40.00)	8 (44.44)	12 (42.86)	
≤5 years old	4 (40.00)	9 (50.00)	13 (46.43)	0.705
>5 years old	6 (60.00)	9 (50.00)	15 (53.57)	
Italian origins	2 (20.00)	3 (16.67)	5 (17.86)	1.000
Foreign origins^1^	8 (80.00)	15 (83.33)	23 (82.14)	
Diagnosis after contact with a case	6 (60.00)	7 (38.89)	13 (46.43)	0.688
Diagnosis in children with symptoms	4 (40.00)	8 (44.44)	12 (42.86)	1.000
Diagnosis during Screening^2^	0 (0.00)	3 (16.67)	3 (10.71)	0.533
BCG^3^ vaccinated	0 (0.00)	1 (5.56)	1 (3.57)	1.000
Not BCG vaccinated	10 (100.00)	17 (94.44)	27(96.43)	
Pulmonary Tuberculosis	8 (80.00)	15 (83.33)	23 (82.14)	1.000
Extrapulmonary Tuberculosis	2 (20.00)	3 (16.67)	5 (17.86)	
Positive TST^4^/Quantiferon	8 (80.00)	17 (94.44)	25 (89.29)	0.284
Negative TST/Quantiferon	2 (20.00)	1 (5.56)	3 (10.71)	
Positive Culture	7 (70.00)	11 (61.11)	18 (64.29)	0.703
Negative Culture	3 (30.00)	7 (38.89)	10 (35.71)	
Positive Microscopy	5 (50.00)	1 (5.56)	6 (21.43)	0.013
Negative Microscopy	5 (50.00)	17 (94.44)	22 (78.57)	

**Table 2:** Characteristics of cases tested with GeneXpert Ultra and p-value evaluating GeneXpert Ultra performances among different groups of children. ^1^ Children born in Italy or abroad to non-Italian parents. ^2^ Children diagnosed during the screening for immigrated and adopted children held by the IDU. ^3^ Bacillus Calmette-Guerin. ^4^ Tuberculin Skin Test.

**Study Children Characteristics**	**Children who Tested Positive in GeneXpert Ultra**	**Children who Tested Negative in GeneXpert Ultra**	**Total**	***p***
	**n = 36 (53.73%)**	**n = 31 (46.27%)**	**n = 67 (%)**	-
Male	26 (72.22)	16 (51.61)	42 (62.69)	0.137
Female	10 (27.78)	15 (48.39)	25 (37.31)	
≤5 years old	17 (47.22)	17 (54.84)	34 (50.75)	0.706
>5 years old	19 (52.78)	14 (45.16)	33 (49.25)	
Italian origins	6 (16.67)	13 (41.94)	19 (28.36)	0.044
Foreign origins^1^	30 (83.33)	18 (58.06)	48 (71.64)	
Diagnosis after contact with a case	14 (38.89)	20 (64.52)	34 (50.75)	0.065
Diagnosis in children with symptoms	20 (55.56)	11 (35.48)	31 (46.27)	0.162
Diagnosis during Screening^2^	2 (5.55)	0 (0.00)	2 (2.98)	0.495
BCG^3^ vaccinated	4 (11.11)	4 (12.90)	8 (11.94)	1.000
Not BCG vaccinated	32 (88.89)	27 (87.10)	59 (88.06)	
Pulmonary Tuberculosis	23 (63.89)	28 (90.32)	51 (76.12)	0.020
Extrapulmonary Tuberculosis	13 (36.11)	3 (9.68)	16 (23.88)	
Positive TST^4^/Quantiferon	35 (97.22)	28 (90.32)	63 (94.03)	0.329
Negative TST/Quantiferon	1 (2.78)	3 (9.68)	4 (5.97)	
Positive Culture	28 (77.78)	9 (29.03)	37 (55.22)	<0.001
Negative Culture	8 (22.22)	22 (70.97)	30 (44.78)	
Positive Microscopy	10 (27.78)	2 (6.45)	12 (17.91)	0.028
Negative Microscopy	26 (72.22)	29 (93.55)	55 (82.09)	

**Table 3:** GeneXpert and GeneXpert Ultra detection rate comparison in children ≤5 years old, children >5 years old, Italian children, and non-Italian children. ^1^ Children born in Italy or abroad to non-Italian parents.

-	**GeneXpert Detection Rate**	**(CI 95%)**	**GeneXpert Ultra Detection Rate**	**(CI95%)**	***p***
≤5 years old	0.308	(0.057-0.559)	0.500	(0.332-0.668)	0.330
>5 years old	0.400	(0.152-0.648)	0.576	(0.407-0.744)	0.413
Italian origins	0.400	(-0.029-0.829)	0.316	(0.107-0.524)	1.000
Foreign origins^1^	0.348	(0.153-0.542)	0.625	(0.488-0.762)	0.053

**Table 4:** GeneXpert and GeneXpert Ultra detection rate comparison in children diagnosed after contact with a case, children with symptoms, children who resulted positive in TST/IGRAs, children who resulted negative in TST/IGRAs, children diagnosed with PTB, and children diagnosed with EPTB. ^1^ Tuberculin Skin Test. ^2^ Interferon-gamma release assays. ^3^ Pulmonary Tuberculosis. ^4^ Extrapulmonary Tuberculosis.

-	**GeneXpert Detection Rate**	**(CI 95%)**	**GeneXpert Ultra Detection Rate**	**(CI95%)**	***p***
Diagnosis after contact with a case	0.462	(0.190-0.732)	0.412	(0.246-0.577)	0.983
Diagnosis in children with symptoms	0.333	(0.067-0.600)	0.645	(0.477-0.814)	0.091
TST^1^/IGRAs^2^ positive	0.320	(0.137-0.502)	0.556	(0.433-0.678)	0.079
TST/IGRAs negative	0.667	(0.133-1.200)	0.250	(-0.174-0.674)	0.486
PTB^3^	0.348	(0.153-0.542)	0.451	(0.314-0.587)	0.563
EPTB^4^	0.400	(-0.029-0.829)	0.813	(0.621-1.004)	0.115

**Table 5:** Number of GeneXpert and GeneXpert Ultra tested on gastric aspirate and other specimens, number of positive results, and tests detection rate.

-	-	**Positive Result**	**Number of Tests**	**Detection Rate**
Gastric Aspirate	GeneXpert	6	14	0.429
	GeneXpert Ultra	25	64	0.391
Other Specimens	GeneXpert	6	26	0.231
	GeneXpert Ultra	17	53	0.321

**Table 6:** Culture and microscopy results in children tested with GeneXpert MTB/RIF.

-	-	**Children who tested positive in GeneXpert**	**Children who tested negative in GeneXpert**	**Total**	***p***
		**n = 10 (%)**	**n = 18 (%)**	**n = 28 (%)**	
Culture	Positive	7 (70.00)	11 (61.11)	18 (64.29)	0.703
	Negative	3 (30.00)	7 (38.89)	10 (35.71)	
Microscopy	Positive	5 (50.00)	1 (5.56)	6 (21.42)	0.013
	Negative	5 (50.00)	17 (94.44)	22 (78.57)	

**Table 7:** Culture and microscopy results in children tested with GeneXpert Ultra.

-	-	**Children who Tested Positive in GeneXpert Ultra**	**Children who Tested Negative in GeneXpert Ultra**	**Total**	***p***
		**n = 36 (%)**	**n = 31 (%)**	**n = 67 (%)**	
Culture	Positive	28 (77.78)	9 (29.03)	37 (55.22)	<0.001
	Negative	8 (22.22)	22 (70.97)	30 (44.78)	
Microscopy	Positive	10 (27.78)	2 (6.45)	12 (17.91)	0.028
	Negative	26 (72.22)	29 (93.55)	55 (8.09)	

## Data Availability

The data and supportive information are available within the article.

## LIST OF ABBREVIATIONS

AAP: American Academy of Pediatrics
BAL: Broncho-Alveolar Lavage
BCG: Bacillus Calmette-Guérin
CI: Confidence Interval
CSF: Cerebrospinal Fluid
CT: Computed Tomography
DST: Drug Sensitivity Testing
EPTB: Extrapulmonary Tuberculosis
GLA: Gastric Lavage Aspirates
IDU: Infection Disease Unit
IGRA: Interferon Gamma Release Assay
IQR: Interquartile Range
LJ: Lowenstein-Jensen
LPA: Line Probe Assay
MDR-TB: Multidrug-Resistant Tuberculosis
MGIT: Mycobacterium Growth Indicator Tube
PCR: Polymerase Chain Reaction
PTB: Pulmonary Tuberculosis
QFT-IT: QuantiFERON-Gold In-Tube
TB: Tuberculosis
TBI: Tuberculosis Infection
TST: Tuberculin Skin Test

## STANDARDS OF REPORTING

STROBE guidelines were followed.
